# Analysis of Material Solutions for Internal Insulation of Masonry Walls—A Case Study

**DOI:** 10.3390/ma18184330

**Published:** 2025-09-16

**Authors:** Marta Pomada, Janina Adamus

**Affiliations:** Faculty of Civil Engineering, Czestochowa University of Technology, 69 Dabrowskiego St., 42-201 Czestochowa, Poland

**Keywords:** building envelope, insulation materials, thermal insulation, thermal properties, interlayer condensation, experimental tests, FEM analysis

## Abstract

The article concerns the internal insulation of a utility room located in the attic of a building from the late 1990s. Due to the freezing of the external wall, an analysis of heat flow through this wall was conducted. Various insulation materials recommended for internal application were tested: EPS and resol board (100 mm thick) and an aerogel mat (10 mm thick). The analyses included the temperature distribution in the wall and indoor thermal conditions. Experimental studies determined the thermal conductivity coefficient (*λ*) of the selected insulation materials and the heat transfer coefficient for the analyzed wall. Numerical analyses were conducted with the TRISCO 12.0w software, which applies the finite element method (FEM), whereas the assessment of interlayer condensation risk was performed using the WUFI^®^ Pro 5.1 program.

## 1. Introduction

One of the key aspects affecting the comfort of living spaces, energy efficiency, and maintenance costs is the thermal insulation of a building’s external partitions. The thermal insulation of partitions depends on the thickness and type of materials used, the quality of construction work, and the presence of thermal bridges, which are points where heat escapes uncontrollably to the outside. Thermal bridges most commonly occur where the continuity of thermal insulation is interrupted, and they significantly reduce the thermal insulation level of the rooms.

The thermal performance of a partition is expressed by the heat transfer coefficient U [W/(m^2^·K)], with lower U-values indicating better insulation. For a layered wall, the U-value can be reduced by increasing the thickness of the insulation layer or by selecting a material with lower thermal conductivity λ.

Newly constructed buildings must meet specific normative requirements. Current requirements for the heat transfer coefficient are specified in the Technical Conditions to be met by buildings and their location [1]. This legal act outlines the normative requirements for newly constructed buildings in Poland, including the current limits for the heat transfer coefficient of building partitions. The regulation is aligned with European Union directives concerning building performance and energy efficiency, ensuring compatibility with broader EU standards. The method of calculating the heat transfer coefficient for various building partitions, including external walls, is specified in the standard EN ISO 6946:2017-10 [2].

Due to the increasing requirements for energy efficiency and low emissions of buildings imposed by the European Union within the Fit for 55 package [3], the demands related to the thermal insulation of partitions are becoming stricter. According to the current regulations [1], the heat transfer coefficient U for external walls of heated rooms where the temperature is maintained at ≥16 °C should not be higher than 0.20 W/(m^2^·K). For the temperature range of 8 ÷ 16 °C (e.g., garages, technical rooms), the maximum U-value should not exceed 0.45 W/(m^2^·K). In unheated rooms, the maximum allowable U-value for external walls is 0.90 W/(m^2^·K).

Unfortunately, many existing buildings do not meet current regulations, making the insulation of external walls a crucial issue. According to design principles, the material of the internal side of the wall should have a high diffusion resistance, while the external side should have a low one to ensure optimal hygrothermal performance. The best solution is to insulate the external side of buildings. However, during the modernization of existing buildings, as noted by Steskens et al. [4], for architectural, aesthetic, or economic reasons, the only possible solution is often internal insulation. Unfortunately, internal insulation systems have two main drawbacks: they do not completely eliminate thermal bridges and reduce internal space.

The challenges waiting to be solved are as follows: the risk of damage to the external wall due to freezing (internal insulation lowers the temperature of the external wall), the need to cope with moisture penetrating from the outside, and the risk of condensation of humid air from the interior on the external wall. Water condensation on the walls can cause the growth of mold and fungi. Therefore, a hygrothermal analysis is necessary to determine the risk of water vapor condensation and to select the appropriate materials and technologies, especially since this concerns many old, often historic buildings that were built in Europe between 1850 and 1960 [5,6], when energy efficiency was not a priority. According to Lu et al. [7], in London alone, traditional buildings (pre-1919) make up 40% of the existing stock. As shown in the work of Amirzadeh et al. [8] this problem also applies to the United States. Additionally, the authors emphasize that for modernizations to be responsible and not cause negative effects for buildings and their inhabitants in the future, research must include forecasting moisture loads, taking into account not only current but also projected rapidly changing climatic conditions.

Despite the risk of moisture problems, internal insulation is becoming an increasingly common choice. In the work of Bjarløv et al. [9], capillary-active (hydrophilic) insulation materials were proposed as a solution to mitigate moisture-related issues. The necessity to protect external walls from getting wet during rainfall was also emphasized.

In situ studies conducted by Freimanis et al. in [10] a historic building, where the temperature and relative humidity between the internal insulation layer and the external wall were monitored, did not show interlayer condensation under the internal insulation or mold growth but revealed the strong susceptibility of the building facade to external weather conditions. Further analyses by Freimanis et al. [11] on the influence of a vapor-open, capillary-active calcium silicate internal insulation system on the hygrothermal behavior of masonry made from various historic bricks showed that, while the temperature distribution was similar across all wall types, significant differences were observed in their moisture content.

In another study by Freimanis et al. [12], the hygrothermal performance of massive masonry walls with 17 interior insulation systems was evaluated under various external boundary conditions, including a steady-state, dynamic dry, wind-driven rain, and drying cycles. The findings indicated that, while the insulation systems demonstrated comparable thermal performance, their moisture behavior varied. Vaportight and vapor-open insulation systems responded differently to the test cycles, depending on the material’s vapor diffusion resistance.

Different internal insulation systems for solid brick walls were also considered by Jensen et al. [13,14]. Jensen et al. [15] conducted a hygrothermal assessment of solid masonry walls with internal insulation with a particular focus on thermal bridges caused by internal partition walls. For Hutkai et al. [16], the actual thermal and moisture conditions of internally insulated walls were also monitored. This time, the studies were conducted to create a numerical model enabling the analysis of the impact of thermal insulation on temperature distribution and moisture accumulation in the external wall and to select the optimal thermal insulation material and system.

Although the studies showed a positive effect of internal thermal insulation on the surface temperature distribution and thermal comfort improvement, there is concern that lowering the temperature of the external masonry may lead to its damage due to freezing when the ambient temperature drops below 0 °C. Aldabibi et al. [17] argue that the impact of adding thermal insulation on the risk of frost damage depends on several factors, the most important of which are brick properties and moisture exposure level. Kočí et al. and Zhouet et al. [18,19] add that moisture resulting from internal insulation of masonry walls can cause biological corrosion of the wood used for building structural elements, especially damage to the ends of wooden beams. Therefore, wall modernization should be accompanied by additional measures that enhance moisture regulation and minimize the risk of conditions leading to wall segment degradation.

The importance of selecting insulation material (thermal conductivity coefficient λ [W/(m·K)]), its thickness, and the location of the thermal insulation was highlighted by Dybowska-Józefiak et al. [20]. They emphasize that the selection of materials should not be random but based on detailed calculations and analyses. Accurate determination of thermal parameters using specialized software allows for reliable estimation of heat loss and temperature distribution, which helps to avoid mistakes not only during the construction phase but also during the building’s operation.

Johansson et al. [21] proposed the use of vacuum panels for internal insulation, which allows achieving the same thermal resistance with a smaller thickness of the insulation layer. U-value calculations showed that internal insulation can significantly reduce energy consumption. However, the lowered temperature in the external wall after adding vacuum panels leads to higher relative humidity in the wooden beams and reduced drying capacity. Jensen et al. [22] examined the impact of different types of interior paints on the hygrothermal performance and the risk of mold growth in internally insulated solid brick walls.

Pagoni and co-authors [6,23] carried out extensive research in Denmark on real buildings internally insulated with various systems, including diffusion-open, diffusion-tight, and capillary-active solutions. The performance of these materials was assessed by monitoring temperature and relative humidity at the interface between the insulation and the wall, and, in some cases, at the ends of wooden beams or within lintels. The studies, conducted over the last decade, considered the influence of both internal and external climatic conditions. The findings indicate that, although internal wall insulation delivers the expected energy savings, areas with thin walls or excessively thick insulation can increase the risk of mold growth. High indoor humidity in combination with a diffusion-open insulation system was identified as the most significant factor contributing to mold development, while other parameters had a lesser impact.

Similar research, taking into account different orientations and levels of repair in a Danish multi-story building, was carried out by Vanhoutteghem et al. [24]. Hamid and co-workers [25], based on field studies in Sweden, indicated that current numerical simulation standards can be a reliable alternative when on-site measurements are not feasible. However, the authors of the work [5] claim that to obtain realistic results from numerical analyses, a three-dimensional analysis is necessary, as a two-dimensional simulation underestimates heat losses by up to 57% compared to a three-dimensional simulation.

Similar field studies were conducted in Southern Europe, considering three climates: Porto, Lisbon, and Bologna [26]. The results indicate that it is beneficial to adopt moderate insulation with moderate thermal resistance. Increasing the thickness of the insulation and its thermal resistance not only does not bring benefits but can also lead to significant negative effects.

Orlik-Kozdroń [27] also believes that to determine the basic moisture parameters, it is necessary to consider the arrangement and structure of masonry elements, as using large insulation thicknesses without considering the properties of the wall cladding (facade) can be negative. All authors emphasize the need for further research on the impact of internal insulation on masonry structures and thermal comfort in the context of climate change. Andreoti et al. [28] emphasize the importance of in situ analysis to enrich the data set, which will allow for the development of new insulation materials and technologies.

In Starakiewicz et al. [29], the authors emphasize the importance of analyzing the development of mold and the risk of water vapor condensation, including on wall surfaces. They presented four equivalent analytical methods that make it possible to identify the causes of these phenomena and prevent the occurrence of mold and condensation. This approach may be particularly important in buildings where for various reasons, retrofitting the partitions or applying external insulation is not feasible (e.g., in many historic buildings under conservation protection).

Research has also begun on the possibility of using biomaterials for internal retrofitting of solid masonry walls [30]. Jensen et al. [31] applied advanced computer models (heat, air, moisture—HAM) to assess the long-term durability of bio-based insulation systems under future climate conditions projected for different emission scenarios between 2020 and 2050, across several locations in Europe.

The cited review of articles indicates that the issues of internal building insulation are currently very relevant. However, most researchers focus on historic buildings, often made of bricks, which are not protected against absorbing moisture from the environment, especially during rainfall. There is a lack of work concerning ordinary residential buildings constructed at the turn of the 20th and 21st centuries when climate issues were not given much attention. At that time, the popularity of coal as a heating material made it commonly believed that it was easier to heat a building than to insulate it from external sources of cold.

The lack of awareness of the necessity to ensure the continuity of thermal insulation meant that the technologies used at that time did not fully protect buildings from the occurrence of thermal bridges, which are a source of heat loss. An example could be the insufficient insulation of the vertical wall of the attic in a single-family house, which was analyzed by the authors of this study.

The aim of this work is to analyze the possibility of performing internal insulation (selecting the optimal material and insulation layer thickness) and to evaluate its impact on temperature distribution within the wall. Conducting such an analysis will allow for a better understanding of the effects of internal insulation, particularly in terms of reducing the risk of surface condensation and improving indoor thermal conditions, which are crucial for the durability and healthiness of residential buildings.

In this work, modern, unconventional insulation materials, such as aerogel mats and phenolic boards, were used. They are characterized by better thermal conductivity compared to standard insulation materials (polystyrene), which allows for the application of a thinner insulation layer while maintaining the same thermal effects. Additionally, these materials have high diffusion resistance, which prevents water vapor from rooms from accessing walls with lower temperatures, preventing condensation and thereby limiting the growth of mold and fungi.

Our work expands the current knowledge by providing experimental data on advanced insulation materials, such as aerogel mats and resol boards, through direct measurement of their thermal conductivity. We also determined the heat transfer coefficient (U-value) of an actual wall, enabling subsequent analyses to be based on real values rather than assumed ones. Combined with thermographic analysis and hygrothermal simulation, these findings bridge the gap between theoretical modeling and empirical validation in the context of retrofitting buildings without external insulation.

## 2. Materials and Methods

**Figure 1:**
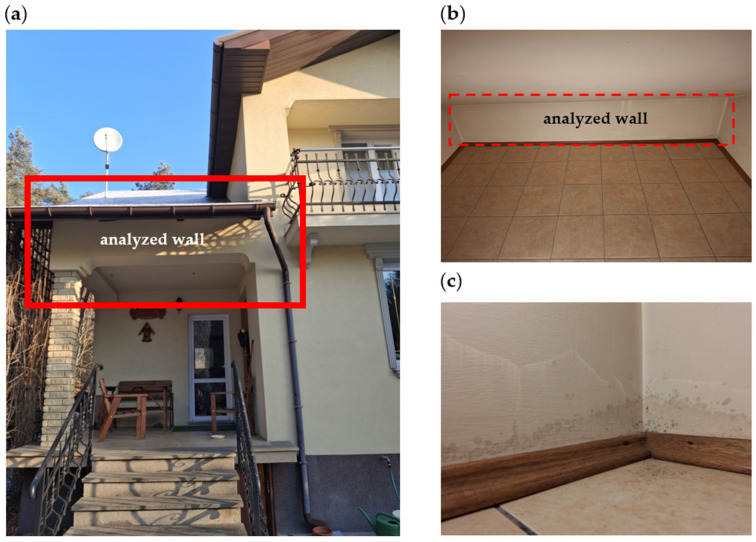
Analyzed wall: (**a**) from the outside, (**b**) from the inside of the room, and (**c**) lower right corner of the room with visible mold. Red rectangles in (**a**,**b**) indicate the area selected for detailed analysis.

This study analyzes the external wall of a room in a building from 1995, located in the usable attic above a terrace (Figure 1). This space is not intended for regular human occupancy; it is primarily used for storage, although it is heated and equipped with a window. The analyzed wall, measuring 0.44 × 2.50 m, is oriented to the south. The external walls of the building are three-layer constructions, made as two parallel walls: the outer wall, 195 mm thick, made of cinder block, and the inner wall, 195 mm thick, is made of ceramic block. The space between the walls is filled with a 60 mm layer of polystyrene. The building’s exterior facade consists of a 15 mm thick layer of cement-lime plaster. On the interior side, the wall is covered with a 15 mm thick layer of gypsum plaster.

During the building’s operation, increasing moisture was observed in the lower corner located on the southwest side. Over time, mold appeared on the wall (Figure 1c). This phenomenon was observed exclusively in the analyzed room, whereas in nearly identical spaces within the same building—constructed using the same technology and materials but oriented differently (east–west and north–west)—no such issue occurred. In these two rooms, the ceiling is located above heated living spaces, which contributes to thermal stability. Measurements confirmed that the analyzed room does not exhibit significant fluctuations in internal temperature or relative humidity that could independently explain the presence of mold.

It is important to note that in the analyzed case, the problematic thermal bridge is not located in the upper corner of the wall adjacent to the roof—this area has been properly insulated. The critical point is the lower corner, where the ceiling rests on an uninsulated reinforced concrete column. The reinforcing steel bars within this column are structurally connected to the load-bearing partition above, creating a hazardous thermal bridge that increases the risk of moisture condensation and degradation of surrounding building materials.

The possibility of insulating the wall from the inside with three insulating materials was analyzed:

- EPS (expanded polystyrene) with a thickness of 100 mm and a declared λ_D_ value of 0.040 W/(m·K), as specified by the manufacturer (Termo Organika Ltd., Cracow, Poland);
- Resol rigid foam insulation board with a thickness of 100 mm and a declared λ_D_ value of 0.021 W/(m·K), as specified by the manufacturer (Kingspan Insulation Limited, Pembridge, Leominster, Herefordshire, UK);
- Aerogel insulation mat with a thickness of 10 mm and a declared λ_D_ value of 0.019 W/(m·K), as specified by the manufacturer (4INSULATION Ltd., Kędzierzyn Koźle, Poland).

Experimental studies were conducted under real conditions from December 2024 to March 2025. The studies included the following:

- Determination of the heat transfer coefficient (U-value) of the analyzed wall using the gOMS II U-value measurement system;
- Temperature distribution on the inner surface of the internal wall using a FLIR E5XT thermal imaging camera (Teledyne FLIR, Wilsonville, OR, USA)

During the experiment, the internal temperature and relative humidity of the room, as well as the external ambient temperature, were also monitored.

Heat flow through the analyzed wall was simulated using the TRISCO 12.0w program [32], while the analysis of interlayer condensation risk was carried out using the WUFI^®^ Pro 5.1 program [33].

**Figure 2:**
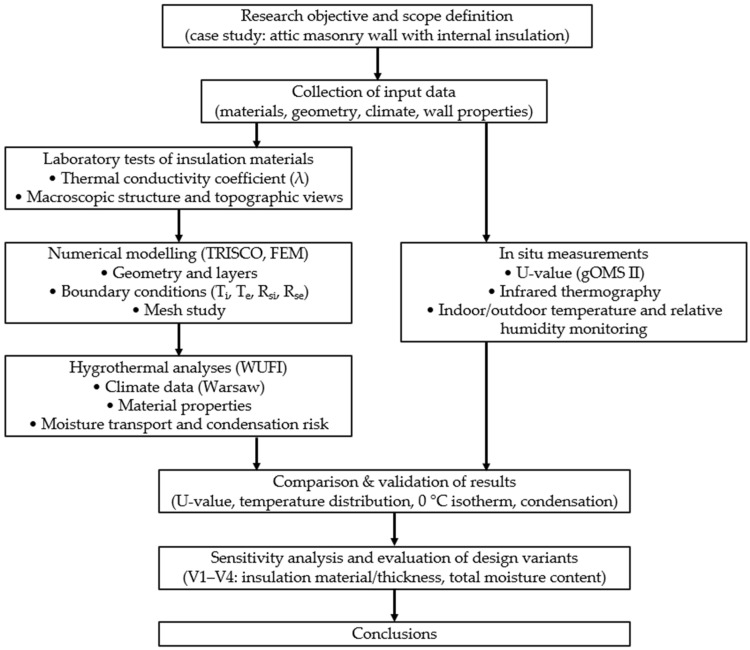
Flowchart of the study showing experimental tests, numerical simulations, result validation, and final conclusions.

To provide a clear overview of the adopted research procedure, a flowchart summarizing the experimental and numerical stages of the study was prepared (Figure 2).

### 2.1. Characterization of Insulation Materials

**Figure 3:**
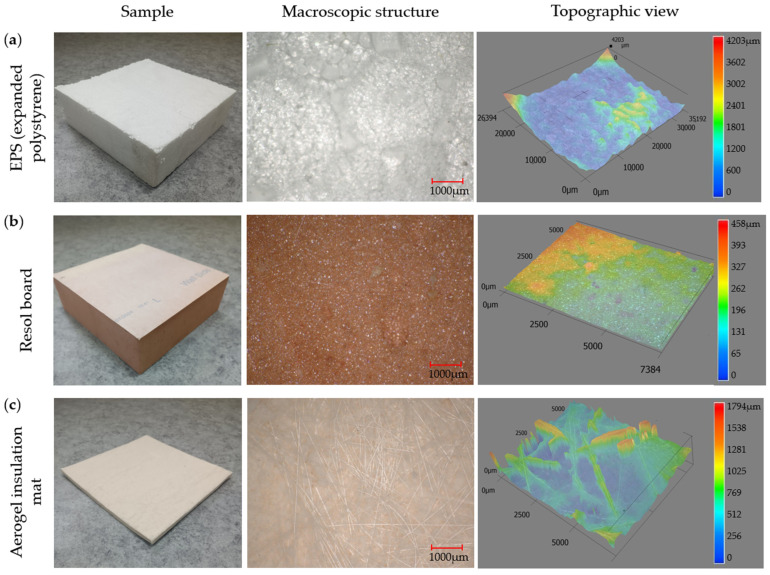
Macroscopic structure and topographic views of analyzed thermal insulation materials: (**a**) EPS (expanded polystyrene), (**b**) resol board, and (**c**) aerogel insulation mat.

The macroscopic structures and topographic views of the analyzed thermal insulation materials (EPS (expanded polystyrene), resol rigid foam insulation board, and aerogel insulation mat) are presented in Figure 3.

The macrostructural analysis of the materials was carried out using the Keyence VHX-900F microscope (Keyence International (Belgium) NV/SA, Mechelen, Belgium) under reflected light. Images of the surfaces of the analyzed materials were taken at 10× magnification. Additionally, a 3D topographic map of the sample surfaces was generated, illustrating their shape, roughness, and potential surface contamination.

In laboratory conditions, the actual thermal conductivity coefficient (λ) of the analyzed insulation materials was determined using a heat flow meter (the NETZSCH HFM 446 Lambda Medium, NETZSCH, Selb, Germany). A detailed description of the λ coefficient measurement is presented in study of Pomada et al. [34].

The dimensions of the samples used for thermal conductivity measurements were 0.305 m × 0.305 m × 0.10 m for the EPS (expanded polystyrene) and resol board, and 0.305 m × 0.305 m × 0.01 m for the aerogel insulation mat. The tests were carried out under the following conditions: the cold plate temperature: −10 °C, the hot plate temperature: 10 °C, the sample temperature: 23 °C, pressure: 2 kPa, the air temperature surrounding the test stand: 20 °C, and ambient relative humidity: 50%.

The thermal conductivity coefficient, denoted as λ_avg_, was determined as an average value from three measurements conducted for each sample in varying orientations. After each test, the sample was either flipped or rotated by 180°. Before measuring the thermal conductivity, the laboratory’s temperature and relative humidity were recorded. The sample dimensions were verified by measuring the lengths of their edges and calculating the mean value. Additionally, the thickness of each sample was measured using a plate apparatus after calibrating the heating plate pressure. To prevent moisture intrusion into the tested insulation materials, the sample edges were sealed with tape.

### 2.2. Methodology for Determining the Heat Transfer Coefficient (U-Value)

To obtain a reliable analysis of the hygrothermal properties of the analyzed wall, a current measurement of its heat transfer coefficient (*U*-value) was conducted using the gOMS II measurement system by greenTeg (greenTEG AG, Zurich, Switzerland) [35]. Determining the U-value based on material characteristics and layer thickness measurements of the wall could result in significant errors, as these properties have likely deteriorated over prolonged use. Furthermore, accurately determining the thermal conductivity coefficients of individual materials is not always feasible. Jensen et al. [36,37] recommend performing in situ measurements.

**Figure 4:**
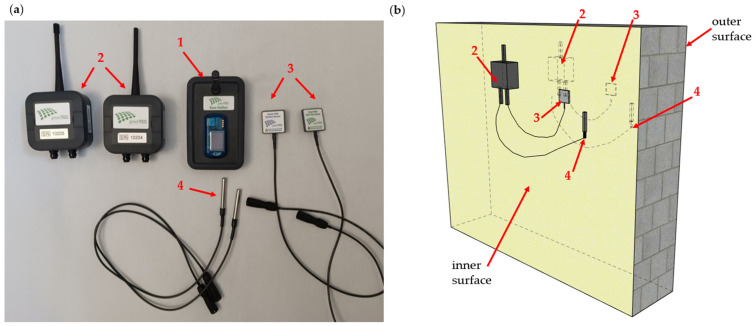
gOMS II measurement set: (**a**) set components 1—base station, 2—measurement nodes, 3—heat flux sensors, 4—air temperature sensors; (**b**) sensor installation diagram.

The gOMS II set includes the following: a base station for data recording, two measurement nodes, an inner combined heat flux and surface temperature sensor, an outer surface temperature sensor, and two ambient air temperature sensors. The entire set is shown in Figure 4.

The measurement method relies on the physical measurement of heat flux and temperatures on the inner surface of the wall, as well as the external and internal temperatures, in accordance with the ISO 9869-1:2014 standard [38], and calculations of the U-value as described in Equation (1). This approach also complies with ASTM C1046 [39] and ASTM C1155 [40] standards.

(1) $$U=\frac{\sum_{j=1}^{n}q_{j}}{\sum_{j=1}^{n}\left(T_{ij}-T_{ej}\right)}$$

where

− q_j_—heat flux at time j [W/m^2^];
− T_ij_—inside air temperature at time j [°C];
− T_ej_—outside air temperature at time j [°C].

Sections 6 and 7 of the ISO 9869-1:2014 standard [38] outline the installation requirements for measurement apparatus and the procedures for analyzing measurement data, respectively. In accordance with the ISO standard, the following key principles for proper installation were met to ensure adequate measurement accuracy:

- Heat flux meters (HFMs) and temperature sensors were placed in locations that accurately represented the entire wall (the measurement site was selected based on a prior inspection using a thermographic camera), avoiding heating or cooling devices, thermal bridges, cracks, or similar elements that could cause measurement errors; external sensors were additionally protected from solar radiation and precipitation;
- The inner and outer sensors were aligned directly opposite each other on the same wall;
- Measurements lasted at least 72 h and during the tests, the temperature difference between the interior and the exterior was maintained at a minimum of 5 °C;
- Data were recorded at 10 min time intervals.

To assess the repeatability of the measurements and enhance the reliability of the results, the U-value measurements were conducted twice, using different positions of heat flux meters (HFMs) and temperature sensors on both the internal and external surfaces. Due to the limited surface area of the analyzed wall and the small number of locations meeting the installation criteria, two independent measurements were performed in the most representative zones.

The results from both measurement sessions showed good agreement and repeatability, with U-value differences falling within the acceptable deviation range of ±10–15%, as specified by the manufacturer and supported by relevant literature.

The gOMS II measurement system allowed for the real-time display of measurement results, enabling continuous monitoring. The measurements were completed when the following conditions were met:

- R-value obtained at the end of the test did not deviate more than 5% from the value obtained 24 h before (dR24);
- R-value obtained during the first 2/3 of the overall measurement period did not deviate more than 5% from the values obtained during the last 2/3 of the overall measurement period.

The measured U-value of the heat transfer coefficient of the wall was utilized in numerical heat flow analyses through the wall and in condensation analysis.

In order to determine the change in thermal parameters of the analyzed external wall, a corrected theoretical value of the heat transfer coefficient (U_C_) was calculated based on the wall construction and the information obtained from the building’s users, in accordance with the EN ISO 6946 standard [2] and Equation (2):

(2) $$U_{C}=U+∆U=U+∆U_{g}+∆U_{f}$$

where

− U_C_—corrected value of heat transfer coefficient [W/(m^2^·K)];
− ΔU_g_—correction for leaks in the thermal insulation layer [W/(m^2^·K)];
− ΔU_f_—correction for mechanical fasteners in the thermal insulation layer [W/(m^2^·K)].

According to the regulation [1], the U_C_ values for walls, roofs, floors, and ceilings, determined in line with European standards and adjusted for corrections, must not exceed the U_C(max)_ limits applicable to all building types. For external walls, with an assumed indoor temperature of T_i_ ≥ 16 °C, the heat transfer coefficient U_C_ must satisfy the following condition:

(3) $$U_{C}\leq U_{C\left(max\right)}=0.20\;W/(m^{2}\cdot K)$$

### 2.3. Methodology for Measuring Wall Surface Temperature

The surface temperature of the analyzed wall was measured using a Flir E5 Pro thermal imaging camera. The images were taken in MSX (Multi-Spectral Dynamic Imaging) thermal mode, which allowed for the display of a thermal image where object edges were enhanced with visual details. The images were edited using the FLIR Thermography software (FLIR Thermal Studio Suite 3.2, Teledyne FLIR, Wilsonville, OR, USA).

To ensure measurement reliability, all thermographic imaging was conducted under controlled conditions. Camera parameters were set according to manufacturer guidelines and real-time environmental data: emissivity ε = 0.95, reflected temperature was 20 °C, ambient temperature was 20 °C, relative humidity was 50%, and a fixed distance of 1 m was between the object and the camera. The average external temperature at the time the images were taken was 3 °C.

The emissivity coefficient was determined using a comparative method with a contact thermometer, following the procedures outlined in ISO 6781-1 [41] and ISO 18434-1 [42]. The camera’s emissivity setting was adjusted until the surface temperature matched the contact thermometer reading. This value was then verified against tabulated data for the tested material from technical literature and manufacturer sources.

Ambient temperature and relative humidity were recorded during each measurement session, as these parameters can influence the accuracy of thermal radiation detection.

**Figure 5:**
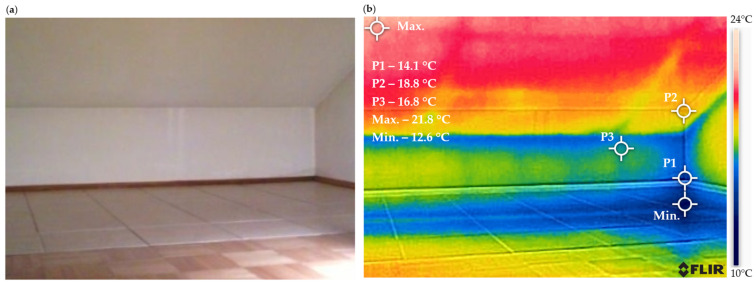
The temperature distribution on the surface of the analyzed wall before thermal modernization: (**a**) actual view and (**b**) thermogram.

Figure 5 shows the temperature distribution on the surface of the analyzed wall.

The thermogram indicates that the lowest temperatures are observed in the lower right corner of the room, in areas where condensation and mold have been detected. The minimum temperature of 12.6 °C was recorded on the floor surface at the junction of the reinforcement elements of the support column and the ceiling. Analysis of the thermogram reveals that the temperature difference is approximately 6 °C.

### 2.4. Numerical Models

Numerical analyses of the temperature distribution in the cross-section and on the inner surface of the analyzed wall variants have been conducted. As part of this analysis, different wall variants were examined to assess their thermal properties.

**Figure 6:**
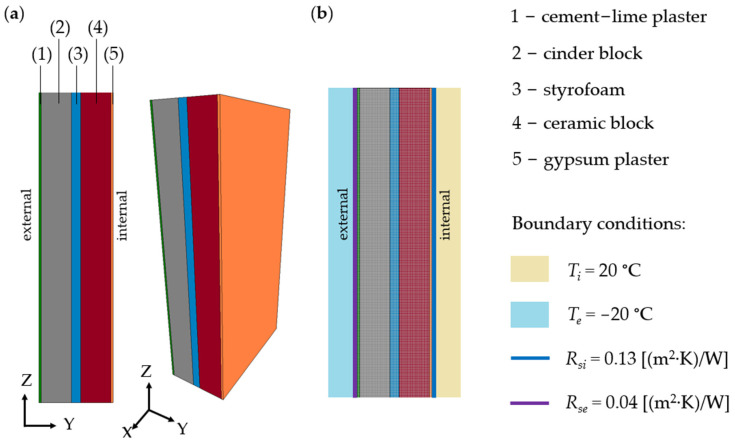
Wall model: (**a**) computational model and (**b**) numerical model.

Variant V1 (baseline) represents the actual state before modernization, consisting of the following layer arrangement: cement-lime plaster (0.015 m), cinder block (0.195 m), styrofoam (0.06 m), ceramic block (0.195 m), and gypsum plaster (0.015 m). In variants V2 to V4, additional thermal insulation layers on the interior side and finishing layers of the wall were included. The detailed layer arrangement for each variant is presented in Table 1 and in Figure 6.

The TRISCO 12.0w program, used for numerical analyses, is based on the finite element method and enables steady-state thermal simulations for orthogonal/rectangular building components in both 2D and 3D. AutoCAD Autodesk 2023 drawings can be imported via DXF files, with material properties (e.g., thermal conductivity and surface emissivity) assigned to specific colors. The software automatically models the equivalent thermal conductivity of air cavities as material properties. Boundary conditions are defined either on surfaces, representing interfaces with the environment or between materials, or within the volume of a material, where constant temperature or heat flux density can be specified. Point boundary conditions are described by fixed temperature or power values.

Calculations were performed under steady-state heat flow conditions, with the following boundary conditions for horizontal heat transfer: thermal resistance on the inner wall surface R_si_ = 0.13 (m^2^·K/W) and on the outer wall surface R_se_ = 0.04 (m^2^·K/W). The internal and external temperatures were set to T_i_ = 20 °C and T_e_ = −20 °C, respectively. These thermal resistance values were adopted in accordance with EN ISO 6946, which provides standardized parameters for building elements with horizontal heat flow. The external surface resistance R_se_ = 0.04 (m^2^·K)/W corresponds to typical outdoor conditions, including an average wind speed of approximately 5 m/s and negligible solar radiation (e.g., overcast weather or measurements taken during early morning or late evening). This assumption is commonly used in thermal simulations to ensure consistency and comparability of results under averaged environmental conditions.

The wall model was represented as a rectangular prism with dimensions of 1.0 m in length and 2.0 m in height, while its width varied depending on the selected wall variant V1 ÷ V4. Material properties were determined based on experimental studies using a lambda meter and measurements of the heat transfer coefficient (U-value).

A numerical grid density of 0.01 m was adopted, resulting in models with the following number of nodes: V1—1,103,531; V2 and V3—1,278,963; and V4—1,136,856. A mesh sensitivity analysis showed that further refinement of the grid resulted in differences of less than 2% in the calculated U-value and temperature distribution, confirming the numerical convergence of the simulations.

Figure 6 presents the computational and numerical models of the V1 wall variant.

For the analyzed wall variants, the temperature distribution was examined, the location of the 0 °C isotherm was determined, and the risk of mold growth on the internal surface of the wall was assessed.

### 2.5. Methodology for Assessing Interstitial and Surface Condensation in Building Partitions

Due to the fact that prolonged exposure to high humidity can lead to the degradation of structural elements and promote the growth of mold, which in turn poses a threat to the health of residents, a detailed analysis of the external wall was conducted to evaluate moisture conditions and their impact on the structural materials. Sustained high moisture content in the partition may lead to the degradation of construction materials. Thermal properties of building materials play a crucial role in this context, as they affect not only heat loss but also the moisture behavior of structural elements. Thermal and moisture processes are closely interrelated—excess moisture contributes to greater heat losses, while temperature changes influence moisture transport. Therefore, both phenomena should be analyzed simultaneously within the framework of hygrothermal studies.

Among the two most commonly used methods for assessing the hygrothermal properties of building envelope components, namely the simplified (steady-state/Glaser) method, described in the ISO 13788 standard [43], and the numerical simulation method (transient models/WUFI), described and regulated by the ISO 15026 standard [44], the latter was chosen.

The WUFI^®^ Pro 5.1 software is specially designed for dynamic simulations of coupled heat and moisture transfer, enabling realistic modeling of hygrothermal conditions in building partitions and entire buildings while taking into account actual climatic conditions. Using WUFI^®^ Pro 5.1 software, one-dimensional hygrothermal calculations were carried out for the cross-section of the analyzed wall, incorporating a built-in database of rain precipitation, humidity, solar and long-wave radiation, capillary transport, and condensation.

The boundary conditions considered in the calculations include the temperature and relative humidity of the indoor and outdoor air, as well as loads associated with precipitation and radiation for the climatic conditions of the city of Warsaw. Since these parameters depend on the inclination and orientation of the analyzed wall, the analyses assumed a southern orientation of the wall S, an inclination of 90°, a low building height (i.e., up to 10 m), and a calculation period of 5 years. Additionally, the typical technological moisture content was assumed for each layer separately, and an initial temperature of 20 °C was established. The indoor climatic conditions were set according to the 3rd class of indoor humidity, as specified by the standard [40]. Moreover, material parameters such as bulk density, porosity, specific heat capacity, thermal conductivity (in a dry state), and the vapor diffusion resistance factor were introduced into the program according to Table 2. The individual layers of the analyzed external wall of the building, along with their thicknesses, were entered into the program. Subsequently, the analyzed component was automatically divided into numerical grid elements, with widths adjusted according to the gradients of temperature and humidity variations.

Hygrothermal analyses were conducted for four variants (V1 ÷ V4) of the analyzed external wall.

According to the ISO 13788 standard [43], the risk of mold growth on the internal surface of the wall should also be assessed by determining the temperature factor f_Rsi_ on the internal surface of the partition. To assess this, the following condition must be verified:

(4) $$f_{Rsi}\geq f_{Rsi(crit)}$$

where

− f_Rsi_—temperature factor at the internal surface of the envelope [–],
− f_Rsi(crit)_—critical value of the temperature factor at the internal surface of the envelope [–].

The temperature factor f_Rsi_ can be calculated using Equation (5) or determined through computational software.

(5) $$f_{Rsi}=\frac{T_{si,min}-T_{e}}{T_{i}-T_{e}}$$

where

− f_Rsi_—temperature factor at the internal surface of the envelope [–];
− T_si,min_—minimum internal surface temperature according to heat flow calculations [°C];
− T_e_—external temperature used in the calculations [°C];
− T_i_—internal temperature used in the calculations [°C].

The critical value of the temperature coefficient f_Rsi(crit)_ was determined for the internal temperature T_i_ = 20 °C and the average monthly relative indoor air humidity of φ_i_ = 50%. In this case, the value of f_Rsi(crit)_ was 0.72.

## 3. Results and Discussion

This section presents the findings of numerical and experimental analyses, focusing on insulation material properties, heat transfer characteristics, thermographic imaging, numerical simulations, and condensation effects. Each aspect is discussed in detail in the following subsections.

### 3.1. Analysis of Insulation Material Properties

Table 3 presents the average thermal conductivity values (λ_avg_) for the analyzed materials, along with essential information about the test and tested materials, such as their mass, density, and measurement duration.

The analysis of experimentally measured thermal conductivity coefficients (λ_avg_) of the tested insulation materials shows that they are nearly identical to the declared thermal conductivity values (λ_D_) provided by the manufacturers. For EPS (expanded polystyrene) and the aerogel mat, the measured values were slightly lower than the declared ones by 0.7% and 1.6%, respectively. In contrast, the resol board showed an average thermal conductivity value that was 2% higher than the declared value. This suggests that for insulation materials stored in dry conditions, the declared values can be used for calculations. However, it should be noted that improper storage conditions significantly deteriorate the thermal conductivity of insulation materials, as discussed by Pomada et al. [34].

### 3.2. Evaluation of U-Value Results

Table 4 presents the adopted material properties and the calculated U_C_ coefficient.

The determined value was compared with the actual *U*-value measured using the gOMS II sensor (greenTEG AG, Zurich, Switzerland).

**Figure 7:**
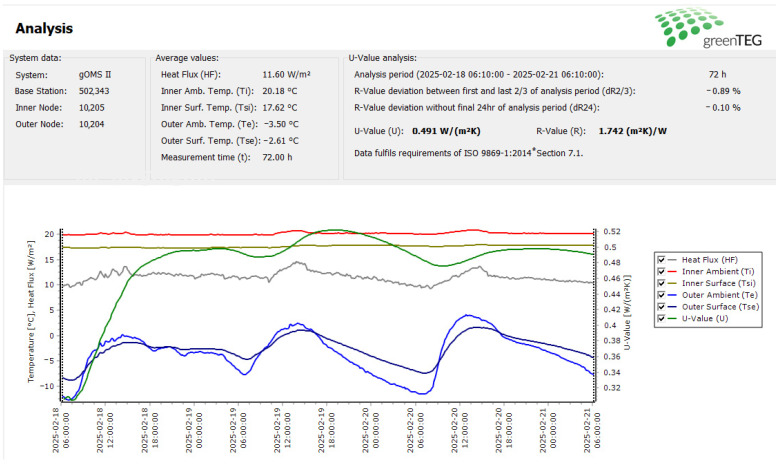
Measurement report of the heat transfer coefficient. *—ISO 9869-1:2014 standard [38].

Figure 7 presents the measurement report for the analyzed wall, which includes the following: average measured temperature values, an analysis of the heat transfer coefficient (U-value), and graphs showing the variations in temperature, heat flux, and the U-value.

During the analyzed measurement period, the average external temperature (T_e_) was −3.5 °C, ranging from −12 °C to 4 °C, with the lowest temperature occurring at night, just before sunrise. The largest temperature fluctuation was recorded between 6:00 AM and 1:00 PM. The average temperature on the external surface of the wall (T_se_) was noted to be −2.6 °C. The indoor temperature was maintained at a constant level, averaging *T_i_* = 20.2 °C, with the temperature on the internal surface of the wall (T_si_) recorded at 17.6 °C. The temperature graphs for both measurements are nearly linear.

The variable external temperature caused fluctuations in the temperature difference on the wall surface, which in turn resulted in variations in the heat flux (HF). The highest heat flux was observed just after noon, when the external temperature began to drop. Changes in the heat flux graph correspond to fluctuations in the external temperature and reflect variability in the U-value graph.

The initial increase in the U-value reflects the stabilization phase of the measurement system (gOMS II), during which sensors reached thermal equilibrium. A rapid rise in outdoor temperature between 06:00 and 12:00 on 18 February caused transient heat flux fluctuations due to the time lag between indoor and outdoor temperatures and slower sensor response to changing external conditions. Once the steady state was achieved (around 19 February, 00:00), the U-value stabilized.

The standard deviation of the thermal resistance (R-value) between the first and last two-thirds of the analysis period (dR2/3) amounted to only −0.89%, while the R-value deviation without the final 24 h of the analysis period (dR24) was −0.10%. This indicates that the conditions of the ISO 9869-1:2014 standard [38] were met, and the measurement results can be considered reliable.

The U-value of the analyzed wall, based on the heat flux measurement, is 0.491 W/(m^2^·K). The U-value measured in situ is 35% higher than the U_C_ value calculated from design data. This indicates that relying solely on the building’s design characteristics could lead to an inaccurate assessment of the building’s thermal performance and heating costs.

### 3.3. Thermographic Image Analysis

**Figure 8:**
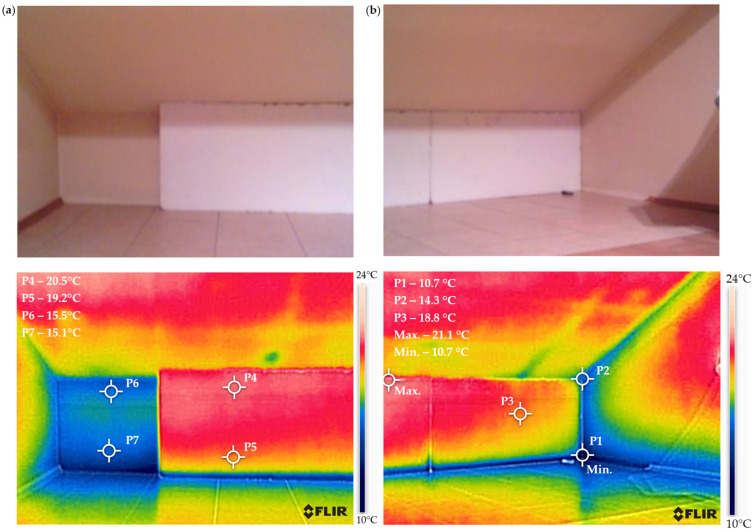
Temperature distribution on the surface of the analyzed wall: (**a**) thermogram of the wall partially insulated internally with EPS (expanded polystyrene) and (**b**) thermogram of the corner on the southwest side, insulated only on the southern wall.

Figure 8 presents the temperature distribution for the wall insulated with EPS (expanded polystyrene). The drawings at the top show the actual view of the wall, and the drawings at the bottom show the thermograms for them.

The insulation of the wall with 100 mm thick polystyrene resulted in a 5-degree increase in the surface temperature from approximately 15 °C at points P6 and P7 on the uninsulated wall to approximately 20 °C at points P4 and P5 on the insulated side. The temperature in the corner measured 10.7 °C at point P1 and 14.3 °C at point P2, respectively. The temperature difference is one of the reasons for water vapor condensation and the appearance of mold fungi, as shown in Figure 1a. The temperature distribution in the corner indicates that insulation should also be applied to the external wall on the western side. A similar temperature distribution was obtained when using 100 mm resol board insulation.

**Figure 9:**
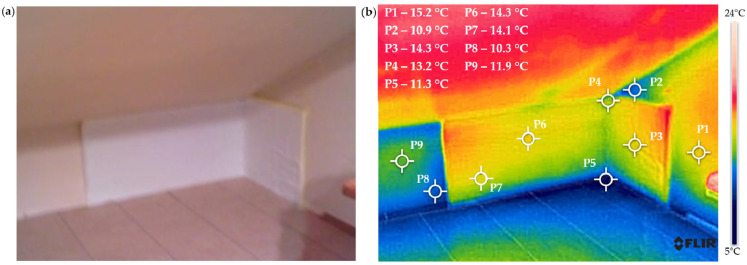
The temperature distribution on the surface of the analyzed wall after partial insulation with a 10 mm aerogel mat: (**a**) actual view and (**b**) thermogram.

Due to the installation capabilities provided by aerogel mats, such as their high flexibility, the insulation was extended to the western side wall. Figure 9 presents a thermographic camera image for this insulation variant.

Despite the significantly smaller thickness (10 mm) of the aerogel mat compared to the thickness of polystyrene and resol board (100 mm), its application resulted in an average increase in the surface temperature of the wall by 3 °C—from 11 °C on the uninsulated surface (points P8, P9) to 14 °C on the surface with the aerogel mat (points P6, P7). There is also no significant difference between the temperature values at the corner points (P4, P5) and the temperature values at the measurement points on the wall’s surface (P1, P3). The use of aerogel mats requires finishing, e.g., with plasterboard, which will certainly further enhance the thermal insulation of the wall.

The analysis of the temperature distribution based on the performed thermograms confirms the justification for insulating the analyzed wall internally using one of the studied materials.

### 3.4. Numerical Analyses

**Figure 10:**
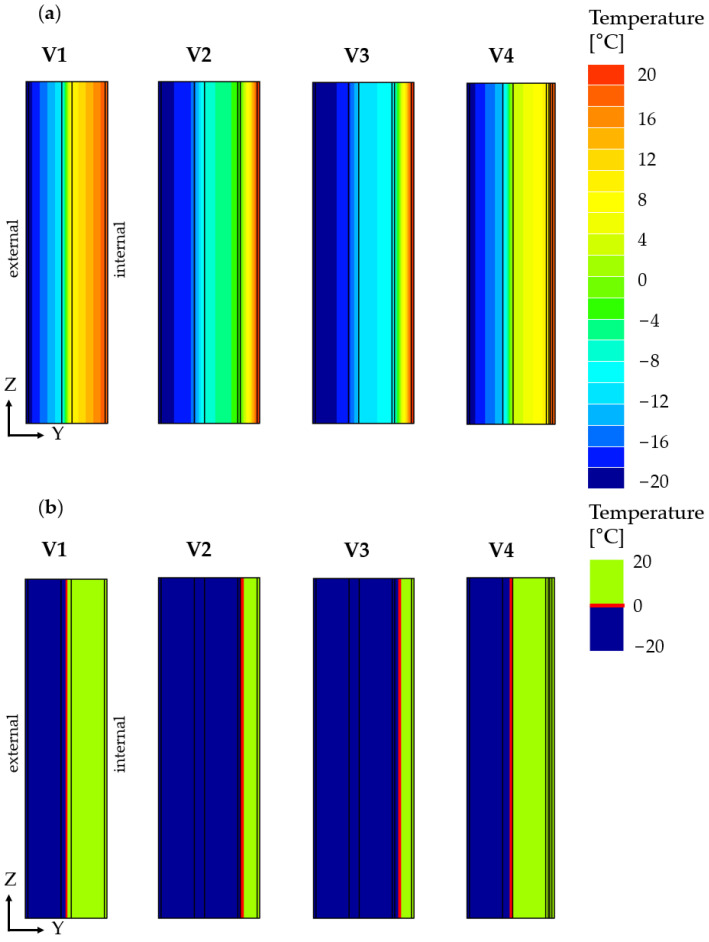
Numerical simulation results: (**a**) temperature distribution in the cross-section and (**b**) position of the 0 °C isotherm.

Figure 10a presents the temperature distribution in the cross-section of each wall variant, while Figure 10b shows the position of the 0 °C isotherm within these walls.

A high density of isotherms is observed in the insulation layers of the analyzed wall variants. In variants V1 and V4, this density occurs in the existing insulation layer located between the cinder block and ceramic block layers. In contrast, in variants V2 and V3, the isotherm concentration is present both in the existing insulation layer and in the additional insulation layer—expanded polystyrene (V2) and resol insulation board (V3), respectively.

The application of an internal thermal insulation layer caused a shift in the 0 °C isotherm deeper into the partition, towards the interior of the building, increasing the area where water freezing can occur in the pores and joints of the load-bearing layer. At the same time, a change in the temperature on the internal surface of the wall was observed. The minimum surface temperature values (T_si,min_) were as follows: 17.48 °C for variant V1, 18.87 °C for V2, 19.24 °C for V3, and 18.05 °C for V4.

It follows that the additional 100 mm thick thermal insulation layer applied in variants V2 and V3 resulted in an average increase of T_si,min_ by approximately 1.5 °C. This is significant in terms of the risk of water vapor condensation on the internal surface of the wall, as well as interstitial condensation within the structure.

Despite the predominantly one-dimensional nature of heat flow shown in Figure 10a,b, TRISCO 12.0w software based on the finite element method (FEM) was selected due to its superior capabilities. The method supports transient analysis under changing weather conditions, accurate modeling of complex geometries and materials, rapid evaluation of alternative design options, and clear graphical outputs such as isotherm and temperature maps. Compared to simplified analytical approaches, FEM offers greater precision, deeper understanding of local thermal effects, and increased flexibility—features particularly valuable when assessing high-performance building envelopes.

### 3.5. Analysis of Interstitial and Surface Condensation

Based on the results of numerical analyses, Condition 4 was verified for all examined cases (V1–V4), demonstrating a complete absence of condensation risk on the internal surfaces of the analyzed external wall variants.

The dynamics of moisture changes in the wall were assessed based on the total water content (kg/m^2^), determined by the mass of moisture per unit area, as well as the analysis of moisture distribution in individual layers of the partition. This allows for the identification of potential risks associated with dampness. Monitoring moisture levels, especially in insulation layers and adjacent layers, is crucial to preventing condensation and mold growth while maintaining the thermal properties of the materials.

The total moisture content in the wall provides information about the dynamics of its transport—whether moisture accumulates or evaporates from the construction materials. An increase in humidity during the analyzed period may indicate a risk of condensation, material degradation, mold growth, frost damage, and deterioration of thermal insulation properties. Conversely, a decrease in water content signifies a drying process, which is essential for the durability and proper functioning of building materials.

The ability of the wall to retain or release moisture was assessed by comparing the initial and final moisture content—the evaluation criterion is met if the final water content is lower than the initial value.

Following the assessment method based on the dryness rate (DR) coefficient presented in Harb et al. [45], an analysis was conducted to evaluate the wall’s ability to dry out and reduce moisture content. The DR coefficient measures the capacity of the partition to release moisture and decrease humidity levels. For each configuration, its value is calculated as the difference between the total initial water content (TWC_i_, kg/m^2^) and the total final water content (TWC_f_, kg/m^2^) in all layers of the analyzed assembly, divided by the total initial water content over a four-year period, as shown in Equation (6).

(6) $$DR=\frac{{TWC}_{i}-{TWC}_{f}}{{TWC}_{i}}\cdot 100$$

where

− DR—dryness rate coefficient [–];
− TWC_i_—total initial water content [kg/m^2^];
− TWC_f_—total final water content [kg/m^2^].

A negative DR coefficient indicates that the wall accumulates moisture throughout the study period, making it particularly susceptible to moisture-related issues. Conversely, a positive DR coefficient suggests that the wall gradually reduces its moisture content. Higher DR values reflect a greater ability to eliminate moisture, whereas lower values indicate a slower rate of moisture reduction.

Table 5 presents the values of TWC_i_, TWC_f_, and DR coefficient for four variants of the external wall, obtained from a five-year simulation.

In all analyzed cases, the DR coefficient is negative, indicating a limited ability of the wall to dry out and leading to moisture accumulation inside. This is particularly evident in variants V2 and V3, where an additional internal 100 mm insulation layer was applied. While this improved the thermal properties of the partition, it also increased its capacity to retain moisture. This behavior can be explained by the material properties: expanded polystyrene and resol boards (V2 and V3) have high vapor diffusion resistance, which limits the drying potential despite improving thermal performance. In contrast, the thin aerogel layer (V4) is more vapor-permeable, which provides a somewhat better balance between insulation and moisture transport.

**Figure 11:**
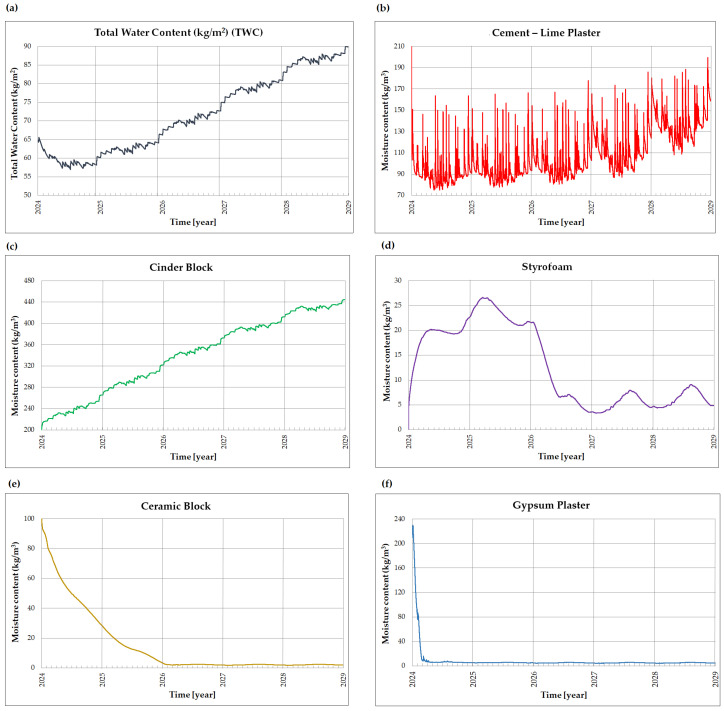
Moisture dynamics in the external wall, variant V1: (**a**) total moisture content (kg/m^2^), (**b**) moisture content in the cement-lime plaster layer (kg/m^3^), (**c**) moisture content in the cinder block layer (kg/m^3^), (**d**) moisture content in the styrofoam layer (kg/m^3^), (**e**) moisture content in the ceramic block layer (kg/m^3^), and (**f**) moisture content in the gypsum plaster layer (kg/m^3^).

To better understand this process, changes in moisture content within individual material layers were analyzed for each wall variant. Figure 11 presents graphs of total moisture content in the wall (a) and moisture distribution across five layers (b–f) for variant V1, representing the existing state.

In the existing wall structure, the cinder block (Figure 11c) accumulates the highest amount of moisture. An analysis of the moisture content graph for the insulation layer indicates that after an initial intense absorption, rapid evaporation occurs, followed by distinct seasonal fluctuations. Maximum moisture levels are observed during cold and humid periods, while minimum values occur in warmer and drier months.

Layers closer to the interior side of the partition, such as the ceramic block (Figure 11e) and the internal plaster (Figure 11f), exhibit nearly zero moisture content throughout the analyzed period after the initial evaporation phase.

**Figure 12:**
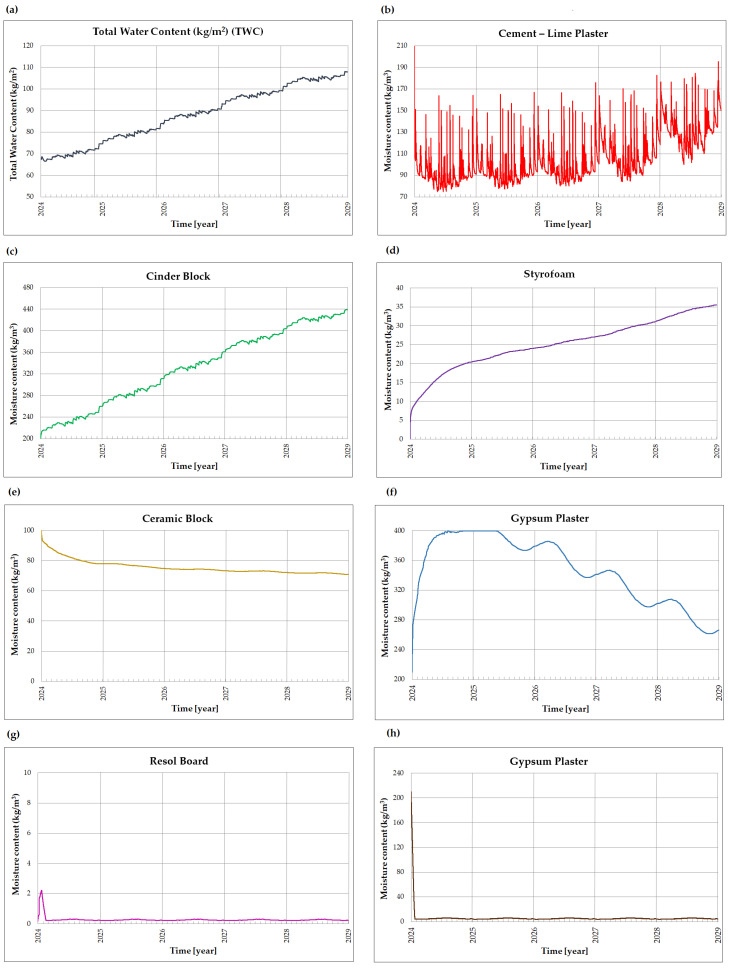
Moisture dynamics in the external wall, variant V3: (**a**) total moisture content (kg/m^2^), (**b**) moisture content in the cement-lime plaster layer (kg/m^3^), (**c**) moisture content in the cinder block layer (kg/m^3^), (**d**) moisture content in the styrofoam layer (kg/m^3^), (**e**) moisture content in the ceramic block layer (kg/m^3^), (**f**) moisture content in the gypsum plaster layer (kg/m^3^), (**g**) moisture content in the resol board layer (kg/m^3^), and (**h**) moisture content in the gypsum plaster layer (kg/m^3^).

The moisture dynamics in the wall after applying internal insulation (variants V2–V4) were also examined. Variants V2 and V3 show almost identical behavior due to the same initial moisture content in the insulating material. Variant V3 (Figure 12) was selected for detailed analysis due to the superior thermal properties of the resol board compared to the styrofoam used in variant V2.

The application of an additional internal thermal insulation layer resulted in increased moisture content in the ceramic block layer (Figure 12e) and gypsum plaster layer (Figure 12f) compared to variant V1. In such a case, it is necessary to consider modifying the layer arrangement in the analyzed wall, including the possibility of introducing an air gap between the existing internal plaster layer and the insulation layer.

**Figure 13:**
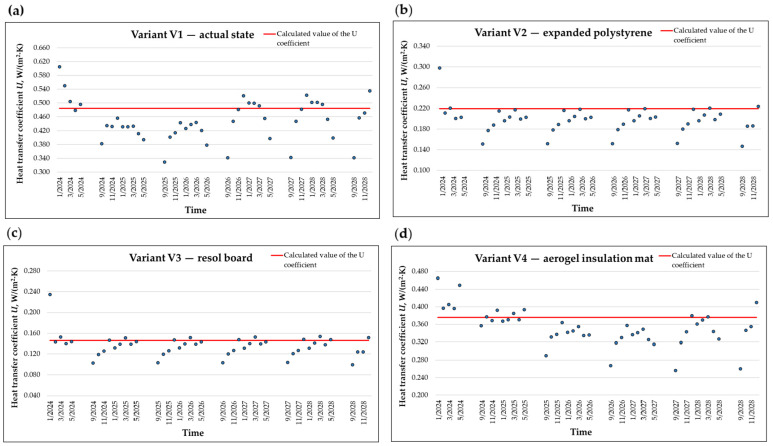
Monthly average variation in the U-value during the heating period for the following: (**a**) variant V1—actual state, (**b**) variant V2—expanded polystyrene, (**c**) variant V3—resol board, (**d**) variant V4—aerogel insulation mat.

Additionally, the impact of moisture in the layers on the thermal properties of the analyzed wall variants should be examined by assessing the heat transfer coefficient U. Figure 13 illustrates the variations in the U-value depending on the period in the calculation cycle.

Summer months were excluded from the analysis of effective thermal transmittance, as during this period the direction of heat flux across the building envelope may reverse multiple times per day. Under dynamic conditions, especially in highly insulated buildings, this can lead to ambiguous results—such as seemingly negative effective U-values—which reflect transient heat balance rather than the material’s physical properties.

The simulation was performed in WUFI^®^ Pro 5.1 for the construction with typical built-in moisture, based on transient results with variable outdoor temperature. The transient thermal transmission postprocessor analyses hourly temperatures and heat flows from the full hygrothermal simulation, thereby accounting for real conditions arising from both weather exposure and occupants’ behavior. This approach also incorporates the effects of variable material properties (particularly the influence of changing moisture content on thermal conductivity), additional thermal transport processes (such as latent heat transport by vapor flows), additional heat sources (such as solar radiation), and parameters dependent on environmental conditions (e.g., wind-dependent surface transfer coefficients).

The calculated thermal transmittance coefficient (U-value) does not fully reflect the actual behavior of the thermal partition under operational conditions.

Based on the analysis of the graphs in Figure 13, a seasonal variation in the U-value is noticeable—the partition exhibits better insulating properties in warmer months, which can be associated with a decrease in moisture content within the material layers. In winter periods, with increased moisture accumulation and the possibility of water vapor condensation, the U-value deteriorates.

This phenomenon confirms the necessity of conducting dynamic hygrothermal analyses to evaluate the energy efficiency of building partitions.

## 4. Conclusions

Improper thermal insulation can increase moisture levels inside the building partition, leading to condensation and mold growth. These phenomena pose risks to both the structural integrity of the building and indoor air quality. Based on the conducted analyses, one of the factors facilitating moisture accumulation in partitions is the arrangement of layers in the wall.

Thermograms and temperature measurements taken at various points on the wall surface, the presence of mold and condensation of water vapor during the winter period, the analysis of interlayer condensation, and the temperature distribution in the partition derived from numerical models confirm the necessity of additional insulation. Due to the presence of an existing external facade, the analysis focused on applying additional insulation from the interior side.

Quantitative results show that applying 100 mm EPS or resol board increased surface temperatures by approximately 5 °C, while a 10 mm aerogel mat raised temperatures by 3 °C. Numerical simulations revealed that the minimum internal surface temperature (T_si,min_) increased from 17.48 °C (uninsulated wall) to 19.24 °C (resol-insulated wall), reducing the risk of surface condensation.

However, dynamic hygrothermal simulations indicated increased moisture accumulation in variants with internal insulation. The dryness rate (DR) coefficient was negative in all cases, with the highest moisture retention observed in EPS and resol variants (DR = −55.96 and −58.80, respectively). This highlights the need to carefully assess moisture transport and retention before implementing internal insulation. Moreover, insulation thickness may also affect hygrothermal behavior, and its impact on moisture accumulation and vapor permeability will be addressed in future research.

The calculated U-value does not fully reflect its real behavior over time, as it undergoes seasonal variations primarily due to moisture content in the partition and fluctuating thermal-humidity conditions. These findings confirm the relevance of dynamic hygrothermal analyses in assessing the energy efficiency of external partitions.

Before implementing internal thermal insulation or conducting an energy efficiency analysis, it is essential to consider the following:

- Measurement of the actual thermal transmittance coefficient of the wall;
- Numerical analyses for the proposed solutions;
- Variable thermal-humidity conditions resulting from water and moisture transport within the opaque wall layers.

Further research will include long-term monitoring using temperature and humidity sensors, transient-state simulations under dynamic conditions, and 3D modeling of moisture behavior in complex geometries. These efforts will support the development of more robust retrofit strategies, especially for historic and mid-20th-century buildings.

## Figures and Tables

**Table 1:** Wall variants analyzed in the WUFI program.

Layer	V2	V3	V4
Baseline (V1)	Baseline (V1)	Baseline (V1)	Baseline (V1)
Internal insulation	EPS (expanded polystyrene) 0.10 [m]	resol insulation board 0.10 [m]	aerogel insulation mat 0.01 [m]
Internal finishing layer	gypsum plaster 0.015 [m]	gypsum plaster 0.015 [m]	drywall 0.0125 [m] + gypsum plaster 0.015 [m]

**Table 2:** Material parameters.

Layer	Bulk Density [kg/m^3^]	Porosity [-]	Specific Heat Capacity [J/kg·K]	Thermal Conductivity [W/m·K]	Vapor Diffusion Resistance Factor [-]	Typical Technological Moisture Content [kg/m^3^]
Cement-lime plaster	1900	0.24	850	1.145	19.00	210.00
Cinder block	1000	0.72	850	0.433	8.30	200.00
Styrofoam	15	0.95	1500	0.063	30.00	0.00
Ceramic block	1744	0.33	889	0.423	10.00	100.00
Gypsum plaster (actual)	850	0.65	850	0.977	8.30	400.00
EPS (expanded polystyrene)	10.7	0.98	1500	0.040	21.70	0.18
Resol insulation board	36	0.98	1500	0.021	35.00	0.18
Aerogel insulation mat	203	0.95	2500	0.019	4.80	8.00
Gypsum plaster (proposed)	850	0.65	850	0.700	8.30	210.00
Drywall	850	0.65	850	0.250	10.00	8.00

**Table 3:** Thermal conductivity for the analyzed materials, including results from own experimental studies and manufacturer-declared values.

Insulation Material	Mass [kg]	Density [kg/m^3^]	Measurement Time of Lambda [min]	Average Test Time [min]	Coefficient λ [W/(m·K)]	Average Coefficient λ_avg_ [W/(m·K)]	Declared Coefficient λ_D_ [W/(m·K)]
EPS (expanded polystyrene)	0.105	10.7	59	63	0.03964	0.03973	0.040
			59		0.03983		
			70		0.03971		
Resol board	0.325	36.0	510	577	0.02140	0.02142	0.021
			567		0.02140		
			654		0.02147		
Aerogel insulation mat	0.197	203.1	54	54	0.01866	0.01870	0.019
			53		0.01882		
			54		0.01861		

**Table 4:** Calculated heat transfer coefficient U_C_ for the analyzed wall.

Layer	Thickness t [m]	Thermal Conductivity Coefficient λ [W/(m·K)]	Thermal Resistance R [(m^2^·K)/W]
Cement-lime plaster	0.015	0.820	0.0183
Cinder block	0.195	0.310	0.6290
Styrofoam	0.06	0.045	1.3333
Ceramic block	0.195	0.303	0.6436
Gypsum plaster	0.015	0.700	0.0214
External thermal resistance R_se_			0.04
Internal thermal resistance R_si_			0.13
Total thermal resistance			2.8157
Heat transfer coefficient U, W/(m^2^·K)			0.3552
Correction for leaks in the thermal insulation layer, ΔU_g,_ W/(m^2^·K)			0.0
Correction for mechanical fasteners in the thermal insulation layer, ΔU_f_, W/(m^2^·K)			0.0072
Corrected heat transfer coefficient U_C_, W/(m^2^·K)			0.3624

**Table 5:** Values of total water content and dryness rate coefficient for the analyzed wall variants.

	V1	V2	V3	V4
Total initial water content TWC_i_ [kg/m^2^]	64.8	67.97	67.97	68.13
Total final water content TWC_f_ [kg/m^2^]	89.85	106.01	107.94	91.42
Dryness rate DR	−38.66	−55.96	−58.80	−34.18

## Data Availability

The original data presented in the study are openly available at: https://cloud.pcz.pl/s/8DfRsJxK4Mf6JQL (accessed on 2 July 2025).

## Nomenclature

ΔU_f_ [W/(m^2^·K)]	correction for mechanical fasteners in the thermal insulation layer
ΔU_g_ [W/(m^2^·K)]	correction for leaks in the thermal insulation layer
DR [–]	dryness rate coefficient
dR2/3 [%]	standard deviation in R-value calculated over the first and last two-thirds of the analysis period
dR24 [%]	standard deviation in R-value without the final 24 h of the analysis period
EPS	expanded polystyrene
ε [–]	surface emissivity
f_Rsi_ [–]	temperature factor at the internal surface of the envelope
f_Rsi(crit)_ [–]	critical value of the temperature factor at the internal surface of the envelope
HF	heat flux
HFMs	heat flux meters
P1, P2, P3, …	measuring points
φ_i_	relative indoor air humidity
q_j_ [W/m^2^]	heat flux at time j
R [(m^2^·K)/W]	thermal resistance
R_se_ [m^2^·K/W]	thermal resistance on the outer wall surface
R_si_ [m^2^·K/W]	thermal resistance on the inner wall surface
t [m]	thickness
T_e_ [°C]	external temperature
T_ej_ [°C]	outside air temperature at time j [°C]
T_i_ [°C]	internal temperature
T_ij_ [°C]	inside air temperature at time j [°C]
T_si_ [°C]	the internal surface of the wall
T_si,min_ [°C]	minimum internal surface temperature
T_se_ [°C]	external surface of the wall
TWC_f_ [kg/m^2^]	total final water content
TWC_i_ [kg/m^2^]	total initial water content
U [W/(m^2^·K)]	heat transfer coefficient
$U_{C}$ [W/(m^2^·K)]	corrected heat transfer coefficient
U_C(max)_ [W/(m^2^·K)]	maximum allowable $U_{C}$ value
V1–V4	analyzed wall variants
λ [W/(m·K)]	thermal conductivity coefficient
λ_avg_ W/(m·K)]	average value from three measurements conducted
λ_D_ [W/(m·K)]	declared thermal conductivity coefficient, specified by the manufacturer
